# Harnessing Fc-Directed Bioconjugation for the Synthesis of Site-Specifically Modified Radioimmunoconjugates

**DOI:** 10.1021/acs.bioconjchem.5c00306

**Published:** 2025-08-09

**Authors:** Camilla Grimaldi, Joni Sebastiano, Wei-Siang Mark Kao, Emilia Strugala, Shane A. McGlone, Tomohiro Watanabe, Tsubasa Aoki, Tomohiro Fujii, Brian M. Zeglis

**Affiliations:** † Department of Chemistry, 5924 Hunter College, The City University of New York, New York, New York 10065-5024, United States; ‡ Ph.D. Program in Biochemistry, Graduate Center of the City University of New York, New York, New York 10016, United States; § Department of Radiology, Memorial Sloan Kettering Cancer Center, New York, New York 10065, United States; ∥ 13381 Ajinomoto Co., Inc., 1-1, Suzuki-Cho, Kawasaki-Ku, Kawasaki-Shi, Kanagawa 210-8681, Japan

## Abstract

A growing body of preclinical and clinical evidence has shown that site-specifically and site-selectively modified immunoconjugates exhibit improved *in vivo* performance compared to their stochastically modified cousins. However, extant approaches to site-specific bioconjugation suffer from a variety of issues that make clinical translation challenging, including instability, complexity, and expense. Herein, we describe a novel chemical approach to the synthesis of site-specifically modified radioimmunoconjugates that is simple and straightforward. To this end, we leveraged an Fc-directed peptide to append free sulfhydryl moieties to unique sites within the Fc domain of the CA19-9-targeting antibody 5B1. These thiols were then modified with phenyloxadiazolyl methylsulfone-bearing variants of the chelator CHX-A″-DTPA, and the immunoconjugate was radiolabeled with [^177^Lu]­Lu^3+^ to produce [^177^Lu]­Lu-DTPA-^PODS^AJICAP-5B1 in high yield, purity, and specific activity. Subsequent analyses confirmed the site-specificity of the modification and demonstrated the high stability and immunoreactivity of the radioimmunoconjugate. Biodistribution studies in athymic nude mice bearing subcutaneous BxPC3 pancreatic cancer xenografts revealed that [^177^Lu]­Lu-DTPA-^PODS^AJICAP-5B1 produced high activity concentrations in tumor tissue as well as high tumor-to-background activity concentration ratios and displayed performance that compared favorably to ^177^Lu-labeled analogues synthesized with traditional stochastic and thiol-selective bioconjugation strategies.

## Graphical abstract

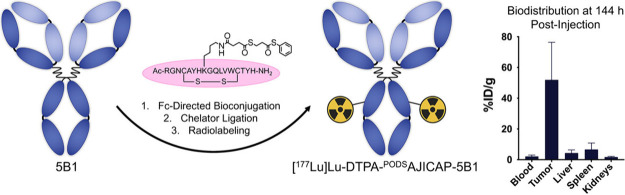

Over the past two decades, immunoconjugates have become indispensable clinical tools for the imaging and treatment of cancer. Indeed, both full length monoclonal antibodies (mAb) and antibody fragments have emerged as effective vectors for the delivery of a wide range of cargoes to tumor tissue.^1^ Historically, immunoconjugates have been synthesized via the stochastic modification of solvent-exposed amino acids — most often lysines — on the surface of the immunoglobulin. Although this approach to bioconjugation is facile and inexpensive, it relinquishes control over the molecular location of the modification as well as the number of cargoes per antibody (i.e., the degree-of-labeling, DOL). As a result, stochastic bioconjugation strategies inevitably produce heterogeneous and poorly defined mixtures of immunoconjugates that can exhibit suboptimal *in vitro* and *in vivo* behavior.^2^

To circumvent these issues, the field has increasingly turned to ‘site-specific’ and ‘site-selective’ methods of bioconjugation designed to attach cargoes to unique sites within immunoglobulins. A variety of innovative and effective approaches to site-specific and site-selective bioconjugation have been developed, including strategies based on unnatural amino acids, click chemistry, enzymatic reactions, the manipulation of the heavy chain glycans, and the modification of the interchain disulfides.^3,4^ Critically, a growing body of preclinical and clinical evidence strongly suggests that these immunoconjugates boast improved *in vivo* performance compared to their stochastically modified cousins.^5–7^ In addition, a recent clinical trial comparing the efficacy of stochastically and site-specifically labeled variants of ^89^Zr-DFO-pertuzumab for the immunoPET of patients with metastatic HER2-expressing malignancies suggests that the latter may produce images with enhanced tumor-to-background contrast.^8^ However, each extant approach to site-specific bioconjugation comes with its own set of scientific, practical, and logistical limitations. Thiol-mediated strategies, for example, are simple and inexpensive but require the reduction of the mAb, produce mixtures of regioisomers, and frequently rely on maleimides that form unstable thioether linkages.^9^ Chemoenzymatic methods, in contrast, eschew reduction and offer more stability but require lengthy incubations with (frequently expensive) enzymes that can pose challenges in the context of clinical production.^10^ Clearly, approaches to site-specific bioconjugation that balance ease, selectivity, and clinical translatability remain an urgent unmet need.

This investigation is predicated on the development of a straightforward and facile approach to the synthesis of site-specifically modified radioimmunoconjugates. To this end, we have harnessed the ‘AJICAP’ reagent: a 17-amino acid cyclic peptide capable of selectively binding the Fc regions of IgG_1_, IgG_2_, or IgG_4_ antibodies and facilitating the site-specific, covalent modification of their K248 residues. More specifically, the reagent recognizes a protein A recognition motif within the Fc domain known as Z34C, thereby positioning a reactive thiophenyl ester in close proximity to the primary amine of K248. Subsequent treatment of the resultant immunoconjugate with hydroxylamine cleaves an alkylthioester bond within the reagent, liberating the peptide and exposing a pair of free sulfhydryl moieties for modification with thiol-reactive probes.^11,12^ Fujii et al. previously illustrated the versatility of this virtually ‘trace-less’ modification technology by creating stable and homogeneous — i.e. DOL = 1.9 ± 0.1 cargoes/mAb — antibody-drug conjugates of trastuzumab and rituximab using a variety of maleimide-bearing toxins (i.e., deruxtecan, maytansinoid, MMAE, MMAF, and tesirine).^11^

Herein, we report the first application of this technology to the synthesis of radioimmunoconjugates. To this end, we have employed a model system with four key components (Figure 1). The immunoglobulin at the heart of the investigation is 5B1, a clinically validated fully human mAb that targets CA19–9, a carbohydrate antigen that is overexpressed in a variety of malignancies but is most widely associated with pancreatic ductal adenocarcinoma (PDAC).^13–15^ Next, we have selected lutetium-177 (^177^Lu; *t*_1/2_ ∼ 6.7 d), a β^–^-emitting isotope frequently employed in radioimmunotherapy, as the radionuclide for this study. Finally, the bifunctional chelator pairs CHX-A″-DTPA (from here on referred to only as ‘DTPA’) — an acyclic chelator that has been used to stably coordinate [^177^Lu]­Lu^3+^ in a wide variety of radiopharmaceuticals — with a phenyloxadiazolyl methyl sulfone (PODS) group that has been shown to selectively, rapidly, and irreversibly react with free sulfhydryl groups.^16^

**Figure 1:**
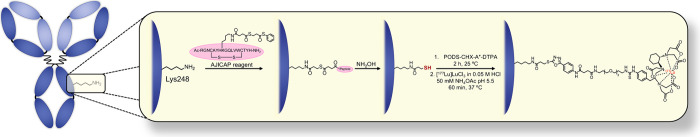
Schematic of the synthesis of [^177^Lu]­Lu-DTPA-^PODS^AJICAP-5B1 using the Fc-binding AJICAP reagent, NH_2_OH, PODS-CHX-A″-DTPA, and ^177^Lu.

The site-specifically modified immunoconjugate - DTPA-^PODS^AJICAP-5B1 - was synthesized in a facile and straightforward manner (Figure 1). First, the AJICAP reagent (5 equiv, of a 20 mM solution in DMF) was added to a solution of 5B1 (5.9 mg/mL in 20 mM sodium acetate buffer, pH 5.5), and the mixture was incubated for 1 h at room temperature. Subsequently, an excess of NH_2_OH•HCl was added, and the solution was allowed to incubate for an additional 1 h. The product of this reaction, ^HS^AJICAP-5B1, was then purified using an NAP-25 desalting column and eluted with phosphate-buffered saline supplemented with 10 mM EDTA (pH 7.4). Purified ^HS^AJICAP-5B1 was then incubated with PODS-CHX-A″-DTPA in phosphate-buffered saline (Chelex-PBS, pH 7.4) for 2 h at RT. The chelator-bearing immunoconjugate was subsequently purified using size-exclusion chromatography, ultimately yielding the final product - DTPA-^PODS^AJICAP-5B1 - in ∼ 65% yield from the parental mAb *(11).*

Two other chelator-bearing variants of 5B1 were also synthesized to facilitate *in vitro* and *in vivo* comparisons (Figure 2A). The first, DTPA-PODS-5B1, is a site-*selectively* modified probe synthesized via the reduction of the disulfide linkages of the mAb with TCEP and the subsequent reaction of the reduced antibody with PODS-CHX-A″-DTPA. The second, DTPA-5B1, is a *stochastically* conjugated immunoconjugate created via the incubation of the antibody with *p*-SCN-Bn-CHX-A″-DTPA under basic conditions. The purity of all three immunoconjugates was verified via SDS-PAGE (Supporting Figure S1), and size exclusion-HPLC of each of the probes revealed low levels (i.e., < 5%) of aggregation (Figure 3A and Supporting Figure S2). MALDI-ToF mass spectrometry was employed to determine the degree of labeling (DOL) of each chelator-bearing immunoconjugate, and the three probes all exhibited similar numbers of chelators per antibody: 2.9 ± 0.6 (DTPA-5B1), 2.8 ± 0.1 (DTPA-PODS-5B1), and 1.8 ± 0.1 (DTPA-^PODS^AJICAP-5B1) (Supporting Table S1).

**Figure 2:**
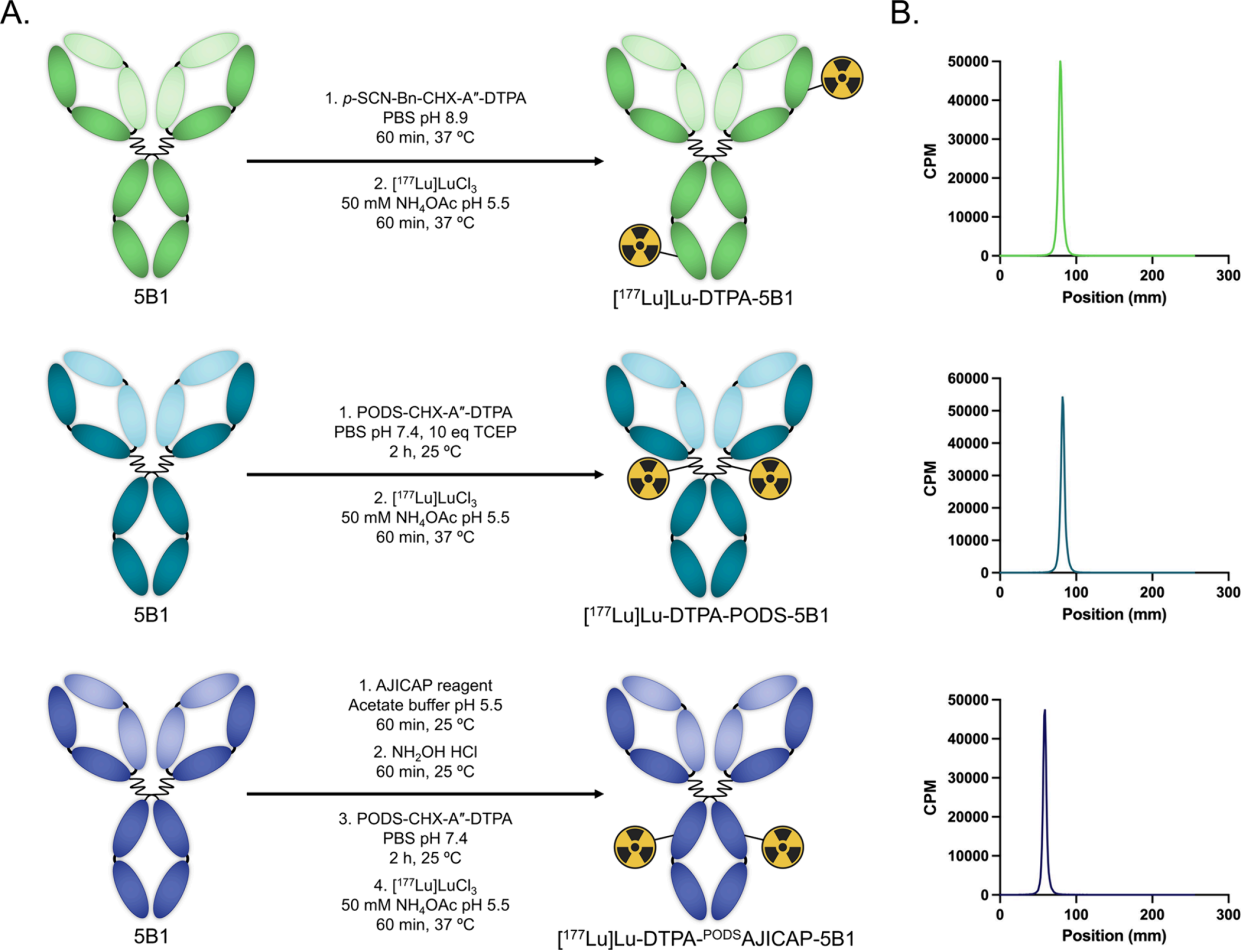
(A) Schematic of the bioconjugation and radiolabeling of [^177^Lu]­Lu-DTPA-5B1 (top), [^177^Lu]­Lu-DTPA-PODS-5B1 (center), and [^177^Lu]­Lu-DTPA-^PODS^AJICAP-5B1 (bottom); (B) radio-instant thin layer chromatograms of purified [^177^Lu]­Lu-DTPA-5B1 (top), [^177^Lu]­Lu-DTPA-PODS-5B1 (center), and [^177^Lu]­Lu-DTPA-^PODS^AJICAP-5B1 (bottom). CPM = counts per minute.

The radiolabeling of each of the immunoconjugates with [^177^Lu]­Lu^3+^ was performed according to standard protocols (Figure 2A). Briefly, the chelator-bearing immunoglobulins (0.6 mg) were incubated with 3.0 mCi [^177^Lu]­LuCl_3_ in 50 mM NH_4_OAc (pH 5.5) for 1 h at 37 °C. After an hour, the progress of the reaction was verified via radio-instant thin layer chromatography; the reaction, if complete, was quenched via the addition of 50 mM EDTA; and the radiolabeled mAb was purified via gel filtration chromatography (Figure 2B). Ultimately, each radioimmunoconjugate — [^177^Lu]­Lu-DTPA-5B1, [^177^Lu]­Lu-DTPA-PODS-5B1, and [^177^Lu]­Lu-DTPA-^PODS^AJICAP-5B1 — was isolated in >95% radiochemical yield, >99% purity, and a specific activity of ∼ 5 mCi/mg (Supporting Table S2). The serum stability of the radioimmunoconjugates was subsequently determined by incubating each ^177^Lu-mAb in human serum for 5 d at 37 °C and periodically analyzing aliquots via radio-ITLC. All three radioimmunoconjugates proved stable to demetalation over this time period, with 92.2 ± 1.5% ([^177^Lu]­Lu-DTPA-^PODS^AJICAP-5B1), 95 ± 1.0% ([^177^Lu]­Lu-DTPA-PODS-5B1), and 96.4 ± 0.2% ([^177^Lu]­Lu-DTPA-5B1) remaining intact over the incubation period (Figure 3C). Next, the immunoreactivities of the ^177^Lu-mAb were assayed via bead-based assay with CA19–9-coated magnetic particles (Figure 3D). All three radioimmunoconjugates displayed immunoreactive fractions >0.75 as well as ‘blockable’ binding of their antigen. Finally, the molecular location of the bioconjugation sites in each conjugate was interrogated via SDS-PAGE of the radioimmunoconjugates followed by autoradiography (Figure 3B). As expected given the location of K248, [^177^Lu]­Lu-DTPA-^PODS^AJICAP-5B1 was radiolabeled exclusively on the heavy chain. Interestingly, however, both [^177^Lu]­Lu-DTPA-PODS-5B1 and [^177^Lu]­Lu-DTPA-5B1 also exhibited radiolabeling predominantly on the heavy chain, though some radioactive signal was associated with the light chain for the latter.

**Figure 3:**
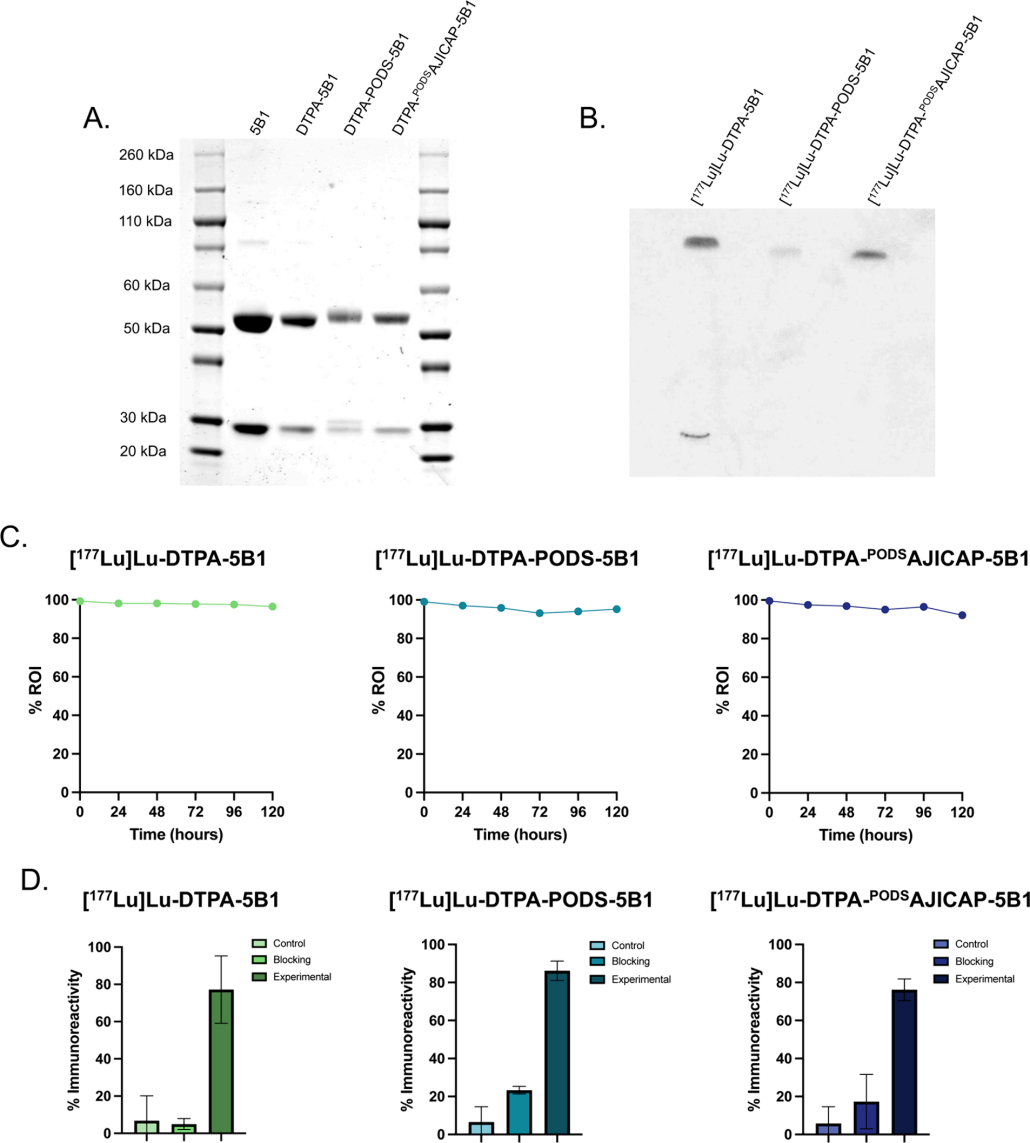
(A) SDS-PAGE of native 5B1, DTPA-5B1, DTPA-PODS-5B1, and DTPA-^PODS^AJICAP-5B1; (B) autoradiography gel of [^177^Lu]­Lu-DTPA-5B1, [^177^Lu]­Lu-DTPA-PODS-5B1, and [^177^Lu]­Lu-DTPA-^PODS^AJICAP-5B1. (C) 5-day serum stability curves of [^177^Lu]­Lu-DTPA-5B1 (left), [^177^Lu]­Lu-DTPA-PODS-5B1 (center), and [^177^Lu]­Lu-DTPA-^PODS^AJICAP-5B1 (right); (D) bead-based immunoreactivity assays of [^177^Lu]­Lu-DTPA-5B1 (left), [^177^Lu]­Lu-DTPA-PODS-5B1 (center), and [^177^Lu]­Lu-DTPA-^PODS^AJICAP-5B1 (right).

With the *in vitro* characterization of the trio of radioimmunoconjugates complete, the next step was to explore their *in vivo* performance in murine models of cancer (Figure 4). To this end, athymic nude mice were inoculated with subcutaneous, CA19–9-expressing BxPC3 pancreatic ductal adenocarcinoma xenografts. Once the xenografts reached ∼100–200 mm^3^, the mice were administered [^177^Lu]­Lu-DTPA-^PODS^AJICAP-5B1, [^177^Lu]­Lu-DTPA-PODS-5B1, or [^177^Lu]­Lu-DTPA-5B1 (100 μCi; 20 μg; in 100 μL 0.9% sterile saline) via the lateral tail vein. At three time points after inoculation — 48, 96, and 144 h (n = 4 mice per radioimmunoconjugate per time point) — the mice were euthanized, and their tumors as well as several other tissues were collected, washed, dried, and assayed for radioactivity on a ^177^Lu-calibrated gamma counter. These biodistribution data clearly showed that all three radioimmunoconjugates exhibited excellent tumor tropism and produced high tumor-to-healthy organ activity concentration ratios. Even at the earliest time point (i.e., 48 h postinjection), [^177^Lu]­Lu-DTPA-^PODS^AJICAP-5B1, [^177^Lu]­Lu-DTPA-PODS-5B1, and [^177^Lu]­Lu-DTPA-5B1 produced tumoral uptake values of 17.9 ± 14.0, 32.5 ± 14.1, and 28.2 ± 14.6 %ID/g, respectively. At the same time point, the healthy tissues with the highest levels of uptake were the blood, liver, and spleen, all with activity concentrations at or below 13 %ID/g. As the experiment progressed, the radioimmunoconjugates cleared from the blood and other healthy tissues while the uptake in the xenografts increased, reaching values of 52.0 ± 24.5, 42.9 ± 13.7, and 18.8 ± 5.0 %ID/g for [^177^Lu]­Lu-DTPA-^PODS^AJICAP-5B1, [^177^Lu]­Lu-DTPA-PODS-5B1, and [^177^Lu]­Lu-DTPA-5B1, respectively, at 144 h postinjection. While some of these values appear to differ, the only differences that are statistically significant is that between [^177^Lu]­Lu-DTPA-5B1 and the two site-selectively modified constructs. At 144 h, the trio of radioimmunoconjugates produced broadly similar tumor-to-blood activity concentration ratios: 24.5 ± 15.6 ([^177^Lu]­Lu-DTPA-^PODS^AJICAP-5B1), 12.9 ± 6.2 ([^177^Lu]­Lu-DTPA-PODS-5B1), and 10.6 ± 16.3 ([^177^Lu]­Lu-DTPA-5B1). However, more variability was apparent in the tumor-to-bone activity concentration ratios at this time point, with [^177^Lu]­Lu-DTPA-^PODS^AJICAP-5B1 (41.2 ± 22.7) higher than [^177^Lu]­Lu-DTPA-5B1 (31.9 ± 9.9) and significantly higher than [^177^Lu]­Lu-DTPA-PODS-5B1 (8.1 ± 3.2) (Supporting Table S3).

**Figure 4:**
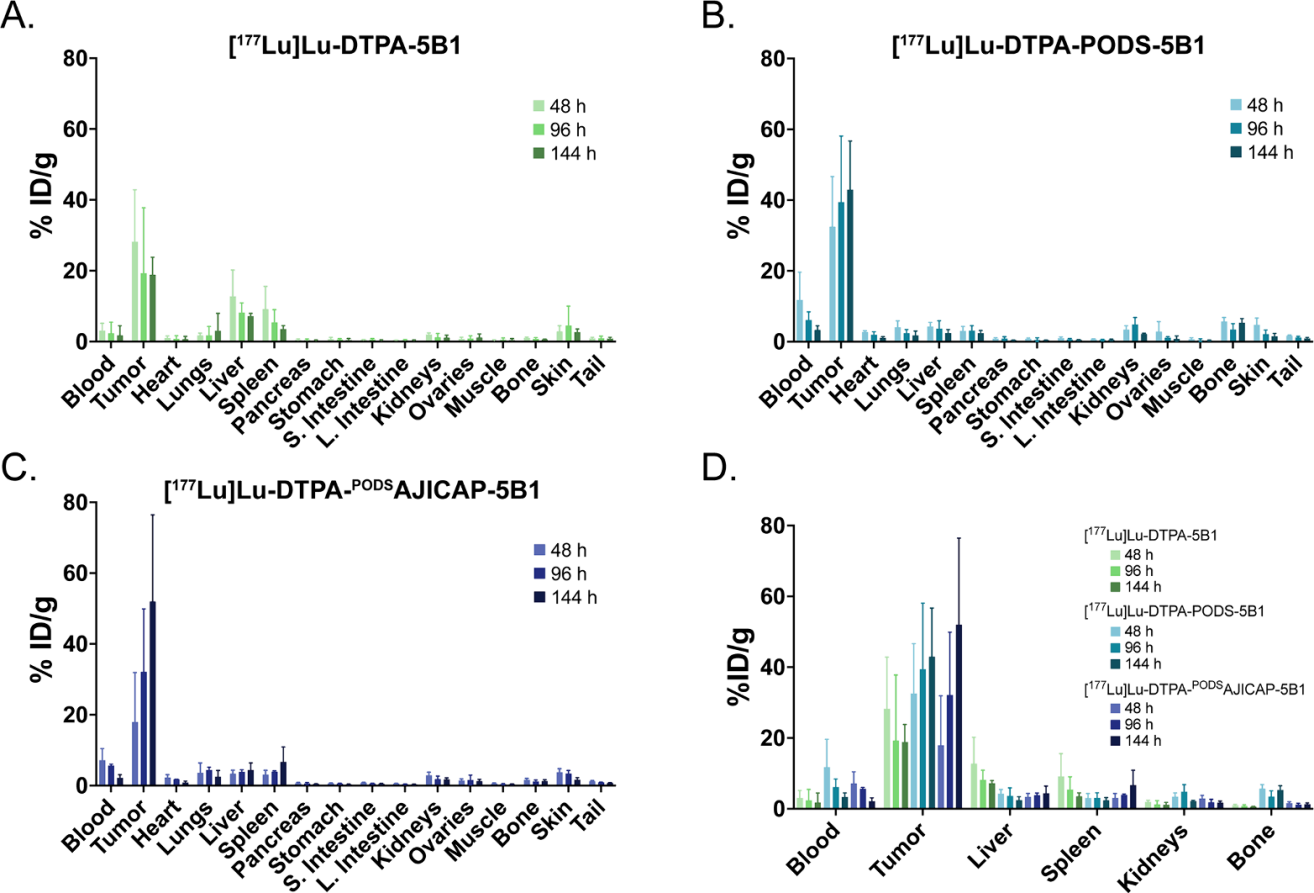
Biodistribution data collected 48, 96, and 144 h after the administration of (A) [^177^Lu]­Lu-DTPA-5B1, (B) [^177^Lu]­Lu-DTPA-PODS-5B1, and (C) [^177^Lu]­Lu-DTPA-^PODS^AJICAP-5B1 to athymic nude mice bearing subcutaneous BxPC3 xenografts; (D) Comparison of the biodistribution data in selected organs.

The chemical and *in vitro* data clearly illustrate that site-specific bioconjugation using the AJICAP reagent facilitates the synthesis of highly homogeneous, well-defined, and stable radioimmunoconjugates. The strength of this approach lies in the fact that it is mild, straightforward — i.e. the initial modification is a simple one-pot-two-step procedure — and purely chemical. Along these lines, the AJICAP technology provides several significant advantages over other extant approaches to site-specific and site-selective bioconjugation. Unlike strategies that rely upon unnatural amino acids, it eschews genetic engineering and can be employed with native antibodies. Unlike chemoenzymatic approaches, it avoids the use of enzymes as well as lengthy incubations. And unlike disulfide-mediated methodologies, it does not require the reduction of the antibody and is site-*specific* rather than site-*selective*. There are also several avenues for the continued development of this technology. As described here, the methodology requires three steps (two of them in a single pot) from antibody to completed immunoconjugate: (*i*) AJICAP conjugation, (*ii*) NH_2_OH deprotection, and (*iii*) thiol-directed ligation. This strategy provides maximum modularity, as the first two yield a sulfhydryl-bearing construct that can react with any bifunctional, thiol-reactive cargo. However, a single-step procedure may also be useful. Indeed, it is possible to envision a one pot procedure in which an AJICAP-type reagent facilitates the site-specific installation of a cargo (i.e., a chelator or toxin) and is then removed to yield the completed immunoconjugate.

The tumor-to-bone activity concentration ratio of [^177^Lu]­Lu-DTPA-^PODS^AJICAP-5B1 at 144 h postinjection was significantly better than that of [^177^Lu]­Lu-DTPA-PODS-5B1, and the former’s tumoral uptake at the same time point was significantly superior to that of [^177^Lu]­Lu-DTPA-5B1. Beyond these values, however, [^177^Lu]­Lu-DTPA-^PODS^AJICAP-5B1 did not yield broadly superior *in vivo* performance compared to the site-selectively and stochastically modified analogues. This is, from our point of view, not necessarily a demerit for the conjugation strategy, as 5B1 is a highly robust antibody that — even with the most haphazard stochastic bioconjugation — already yields high tumoral uptake and tumor-to-healthy organ activity concentration ratios. It is possible (even likely) that the *in vitro* and *in vivo* benefits of this site-specific bioconjugation will be more apparent with more fragile mAb. To explore this hypothesis, we are currently working to develop AJICAP-modified radioimmunoconjugates with several less optimized immunoglobulins.

In sum, this investigation demonstrates that the AJICAP reagent facilitates the straightforward synthesis of homogeneous, well-defined, and stable radioimmunoconjugates with excellent *in vivo* performance. We are currently working to simplify this approach, demonstrate its modularity across antibodies and chelators, and adapt the strategy for automation.

## Supplementary Material



## Notes

Experimental procedures, SE-HPLC chromatograms, SDS-PAGE results, tables of MALDI-ToF data, and tables of radiolabeling and biodistribution data (PDF)

## Abbreviations

DTPA: diethylenetriaminepentaacetic acid
AJICAP: Ajinomoto Fc-directed cyclic peptide
CA19–9: carbohydrate antigen 19–9
Mal: maleimide
TCEP: tris­(2-carboxyethyl) phosphine
DOL: degree-of-labeling
mAb: monoclonal antibody
ADC: antibody-drug conjugate
K248: lysine 248
PDAC: pancreatic ductal adenocarcinoma
SDS-PAGE: sodium dodecyl sulfate-polyacrylamide gel electrophoresis
MALDI-ToF: matrix-assisted laser desorption/ionization-time-of-flight mass spectrometry
SE-HPLC: size exclusion high performance liquid chromatography
PODS: phenyloxadiazolyl methyl sulfone
%ID/g: % of injected dose per gram
PBS: phosphate-buffered saline
DMSO: dimethyl sulfoxide
DMF: dimethylformamide
RPM: revolutions per minute
EDTA: ethylenediaminetetraacetic acid
iTLC: instant thin-layer chromatography

## References

[1] Lin M., Paolillo V., Le D. B. (2021). Monoclonal antibody based radiopharmaceuticals
for imaging and therapy. Curr. Probl Cancer.

[2] Massa S., Xavier C., Muyldermans S., Devoogdt N. (2016). Emerging site-specific
bioconjugation strategies for radioimmunotracer development. Expert Opinion on Drug Delivery.

[3] Adumeau P., Sharma S. K., Brent C., Zeglis B. M. (2016). Site-Specifically
Labeled Immunoconjugates for Molecular Imaging — Part 1: Cysteine
Residues and Glycans. Molecular Imaging and
Biology.

[4] Adumeau P., Sharma S. K., Brent C., Zeglis B. M. (2016). Site-Specifically
Labeled Immunoconjugates for Molecular Imaging — Part 2: Peptide
Tags and Unnatural Amino Acids. Molecular Imaging
and Biology.

[5] Vivier D., Fung K., Rodriguez C. (2020). The Influence of Glycans-Specific
Bioconjugation on the FcγRI Binding and In vivo Performance
of 89Zr-DFO-Pertuzumab. Theranostics.

[6] Agarwal P., Bertozzi C. R. (2015). Site-Specific Antibody–Drug
Conjugates: The
Nexus of Bioorthogonal Chemistry, Protein Engineering, and Drug Development. Bioconjugate Chem..

[7] Kristensen L. K., Christensen C., Jensen M. M. (2019). Site-specifically labeled
89Zr-DFO-trastuzumab improves immuno-reactivity and tumor uptake for
immuno-PET in a subcutaneous HER2-positive xenograft mouse model. Theranostics.

[8] Yeh R., O’Donoghue J., Jayaprakasam V. S. (2024). First-in-Human Evaluation
of Site-Specifically Labeled 89Zr-Pertuzumab in Patients with HER2-Positive
Breast Cancer. Journal of Neclear Medicine.

[9] Shen B.-Q., Xu K., Liu L. (2012). Conjugation site modulates the in vivo stability
and therapeutic activity of antibody-drug conjugates. Nat. Biotechnol..

[10] Rodriguez C., Delaney S., Sebastiano J. (2023). Site-selective radiolabeling
using mushroom tyrosinase and the strain-promoted oxidation-controlled
1,2-quinone cycloaddition. RSC Adv..

[11] Fujii T., Matsuda Y., Seki T. (2023). AJICAP Second Generation:
Improved Chemical Site-Specific Conjugation Technology for Antibody–Drug
Conjugate Production. Bioconjugate Chem..

[12] Watanabe T., Fujii T., Stofleth J. T. (2023). Scale-Up Synthesis of
Site-Specific Antibody–Drug Conjugates Using AJICAP Second-Generation
Technology. Organic Press Research and Development.

[13] Haglund C., Roberts P., Kuusela P. (1986). Evaluation of CA 19–9
as a serum tumour marker in pancreatic cancer. Br. J. Cancer.

[14] Sawada R., Sun S.-M., Wu X. (2011). Human Monoclonal Antibodies
to Sialyl-Lewisa (CA19.9) with Potent CDC, ADCC, and Antitumor Activity. Clin. Cancer Res..

[15] Rodriguez C., Sarrett S. M., Sebastiano J. (2024). Exploring
the Interplay Between Radioimmunoconjugates
and Fcγ Receptors in Genetically Engineered Mouse Models of
Cancer. ACS Pharmacology and Translational Science.

[16] Adumeau P., Davydova M., Zeglis B. M. (2018). Thiol-Reactive
Bifunctional Chelators
for the Creation of Site- Selectively Modified Radioimmunoconjugates
with Improved Stability. Bioconjugate Chem..

