# Supplementary material for: Harnessing Fc-Directed Bioconjugation for the Synthesis of Site-Specifically Modified Radioimmunoconjugates
Source: Bioconjug Chem. 2025 Aug 9;36(8):1588–94. doi: 10.1021/acs.bioconjchem.5c00306 (PMC12371682; doi:10.1021/acs.bioconjchem.5c00306)
Supplement: Supplementary file 1 [file bc5c00306_si_001.pdf]

<u>*Supporting Information*</u>

Harnessing Fc-Directed Bioconjugation for the Synthesis of Site-Specifically Modified Radioimmunoconjugates

Camilla Grimaldi*[1-3], Joni Sebastiano*[1-3], Wei-Siang Mark Kao[1,3], Emilia Strugala[1], Shane A. McGlone[1], Tomohiro Watanabe[4], Tsubasa Aoki[4], Tomohiro Fujii[4], Brian M. Zeglis[1-3]

[1] Department of Chemistry, Hunter College, City University of New York, New York, New York

[2]Ph.D. Program in Biochemistry, Graduate Center of City University of New York, New York, New York

[3]Department of Radiology, Memorial Sloan Kettering Cancer Center, New York, New York

[4]Ajinomoto Co., Inc., 1-1, Suzuki-Cho, Kawasaki-Shi, Kanagawa 210-8681, Japan

*These authors contributed equally to this work

Contents

Supplementary Materials and Methods……………………………………………………………….2

Supplementary Figures……………………………………………………………………………...5

Supplementary Tables………………………………………………………………………………7

Supplementary Materials and Methods

Safety Statement

The handling of radioactivity carries inherent risks. During this investigation, we worked with our institution's Office of Radiation Safety to ensure that proper equipment and procedures were in place to keep radiation exposure levels in line with As Low as Reasonably Achievable (ALARA) standards.

Instrumentation

All instruments were calibrated and maintained according to the standard quality control practices and procedures. UV-vis measurements were taken on a NanoDrop One microvolume UV–vis spectrophotometer (ThermoFisher Scientific; Waltham, MA). Radioactivity measurements were taken using a CRC-15R dose calibrator (Capintec; Ramsey, NJ) and an automatic Wizard2 gamma counter (PerkinElmer; Waltham, MA).

Cell Culture

The human pancreatic adenocarcinoma cell line BxPC-3 (CRL-1687) was purchased from the American Type Culture Collection and authenticated by the Specimen Processing/Research Cell Bank Shared Resource using the short tandem repeat combined DNA Index System typing. Cells were maintained using RPMI-1640 Medium supplemented with 10% fetal calf serum in an incubator at 37 °C and 5% $CO_{2(g)}$. Cells were passaged upon reaching 80% confluency with Gibco TrypLE Express enzyme (1×) with phenol red (ThermoFisher) for 5 min. The cells were also strained using cell strainers (MACS SmartStrainers, 30 and 70 µm; Miltenyi Biotec; Auburn, CA) to ensure a homogeneous cell suspension prior to any experiments or xenografting.

Synthesis of Immunoconjugates

Native 5B1 was prepared in filtered Chelex 100-treated (Bio-Rad Laboratories; Hercules, CA) phosphate-buffered saline (Chelex-PBS, pH 7.4) to final concentrations of 1 mg/mL. Stochastically labeled DTPA-5B1 was synthesized by adjusting the pH of the antibody solution (1 mg, 1 mL) to 8.9 with 0.1 M Na_2CO_3 and then slowly adding 15 equiv. (7 µL) of 10 mg/mL *p*-SCN-Bn-CHX-A"-DTPA in DMSO. This mixture was then incubated at 37 °C and 500 rpm for 1 h. DTPA-PODS-5B1 was synthesized by adding 10 equiv. (13.3 µL) of TCEP and 25 equiv. (19 µL) of 10 mg/mL PODS-CHX-A"-DTPA to the antibody solution (1 mg, 1 mL) and incubating for 2 h at 25 °C and 500 rpm. All conjugates were purified via size-exclusion chromatography (PD-10 column; GE Healthcare; Chicago, IL, USA) and concentrated using 2 mL Amicon Ultra centrifugal filters with a 50 kDa molecular weight cutoff (MilliporeSigma). DTPA-^{PODS}AJICAP-5B1 was synthesized according to published protocols as described in the main manuscript.

SDS-PAGE and Autoradiography Gels

To evaluate the stability and purity of the immunoconjugates and to confirm the site-specific labeling of [^{177}Lu]Lu-DTPA-^{PODS}AJICAP-5B1, two different gels were run: (i) an SDS-PAGE gel of 5B1, DTPA-5B1, DTPA-PODS-5B1, and DTPA-^{PODS}AJICAP-5B1; and (ii) an autoradiography gel of all immunoconjugates and [^{177}Lu]Lu-DTPA-5B1, [^{177}Lu]Lu-DTPA-PODS-5B1, and [^{177}Lu]Lu-DTPA-^{PODS}AJICAP-5B1. Unlabeled, or 'cold', samples were prepared by adding 5 µg of each antibody with LDS loading buffer (12.5 µL), reducing buffer (5 µL), and MilliQ H_2O to a total volume of 50 µL. Radioactive samples were prepared by adding 5 µg (2.5 µCi) of each radioimmunoconjugate with LDS loading buffer (12.5 µL), reducing buffer (5 µL), and MilliQ H_2O to a total volume of 50 µL. All samples were denatured with heat on a Thermomixer set to 90 °C and mixed for 10 min at 500 rpm. Samples were loaded onto a NuPAGE™ 4-12% Bis-Tris gel and run in MOPS SDS Running Buffer. A 10 µL sample of Novex Sharp Pre-Stained Protein Ladder was loaded to the outermost wells. The gel was run at 80 V until the lowest molecular weight ladder band reached the bottom of the gel. The gel was washed 3× with MilliQ H2O, stained with SimplyBlue™ SafeStain (Thermofisher) for 60 min, and washed 3× more with MilliQ H2O. The gel was imaged using a LI-COR Odyssey® CLx instrument and analyzed using Image Studio™ Acquisition Software. After imaging, the autoradiography gel was wrapped in cellophane and placed in a FisherBioTech Autoradiography Cassette with a phosphor imaging plate atop the gel. The cassette was incubated in the dark for 24 h, after which the plate was imaged with a GE Typhoon FLA 7000 instrument and analyzed with Typhoon FLA 7000 Control Software.

Immunoreactivity Assays

The immunoreactive fractions of [^{177}Lu]Lu-DTPA-^{PODS}AJICAP-5B1, [^{177}Lu]Lu-DTPA-PODS-5B1, and [^{177}Lu]Lu-DTPA-5B1 were determined via a bead-based assay. Briefly, 40 µL of Dynabeads™ MyOne™ Streptavidin T1 beads were aliquoted per sample and placed on an Invitrogen™ DynaMagTM-2 Magnetic Rack to isolate the beads from the storage buffer. The storage buffer was discarded, and the beads were washed 2× with PBS supplemented with 0.05% Tween 20 (PBS-T). 1.5 µg of 3'-Sialyl Lewis A-PAA-biotin antigen was added to the beads, and the resulting mixture was agitated and allowed to react on a ThermoFisher Scientific Tube Revolver Rotator at room temp for 30 min. After this incubation, the beads were again washed 2× with PBS-T, and 1 ng of radioimmunoconjugate was added to each sample. Again, the tubes were incubated for 30 min on the rotator. After this last incubation, tubes were placed on the magnetic rack, and the supernatants were collected in separate tubes. Lastly, two final washes of the beads with PBS-T were performed and collected in separate tubes. Two control cohorts, consisting of a blocking group that received 50 µg of cold immunoconjugate and a control group with no antigen added,

were used alongside each experimental cohort. The amount of radioactivity in each sample was analyzed on a ^{177}Lu-calibrated gamma counter, and the activities (counts/minute) were background- and decay-corrected to the start of the run. The immunoreactive fraction was determined by dividing the amount of radioactivity associated with the beads by the total radioactivity in the beads, supernatant, and wash samples.

Animal Care

All animal care was approved by the Institutional Animal Care and Use Committees (IACUCs) of Hunter College and Weill Cornell Medical College. Five to seven-week-old athymic nude mice were obtained from The Jackson Laboratory (Bar Harbor, ME, USA) and allowed to acclimatize for 1 week prior to inoculation. The animals were housed in ventilated cages and given food and water ad libitum. Prior to inoculation, the mice were anesthetized by inhalation of 2% isoflurane/oxygen gas mixture (Baxter Healthcare; Deerfield, IL, USA), and the injection site was sanitized with an ethanol wipe. Tumors were induced in the right shoulder via the subcutaneous injection of 5×10^6 BxPC-3 cells in a 1:1 mixture of media:MatriGel (Corning Life Sciences; Corning, NY, USA).

Supplementary Figures

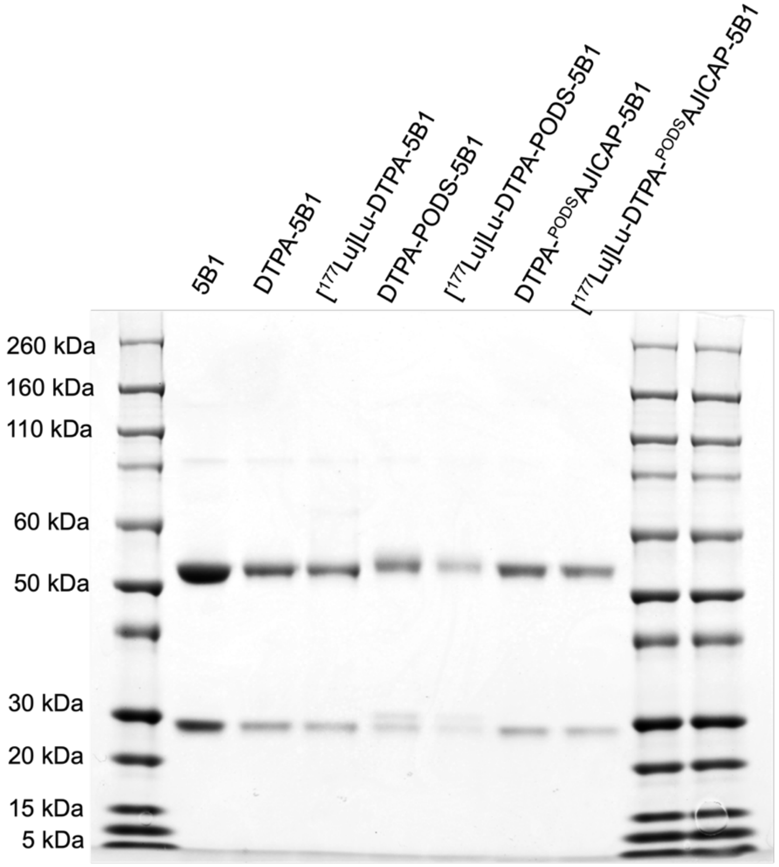

Figure S1. SDS-PAGE gel of 5B1, DTPA-5B1, [^{177}Lu]Lu-DTPA-5B1, DTPA-PODS-5B1, [^{177}Lu]Lu-DTPA-PODS-5B1, DTPA-^{PODS}AJICAP-5B1, and [^{177}Lu]Lu-DTPA-^{PODS}AJICAP-5B1.

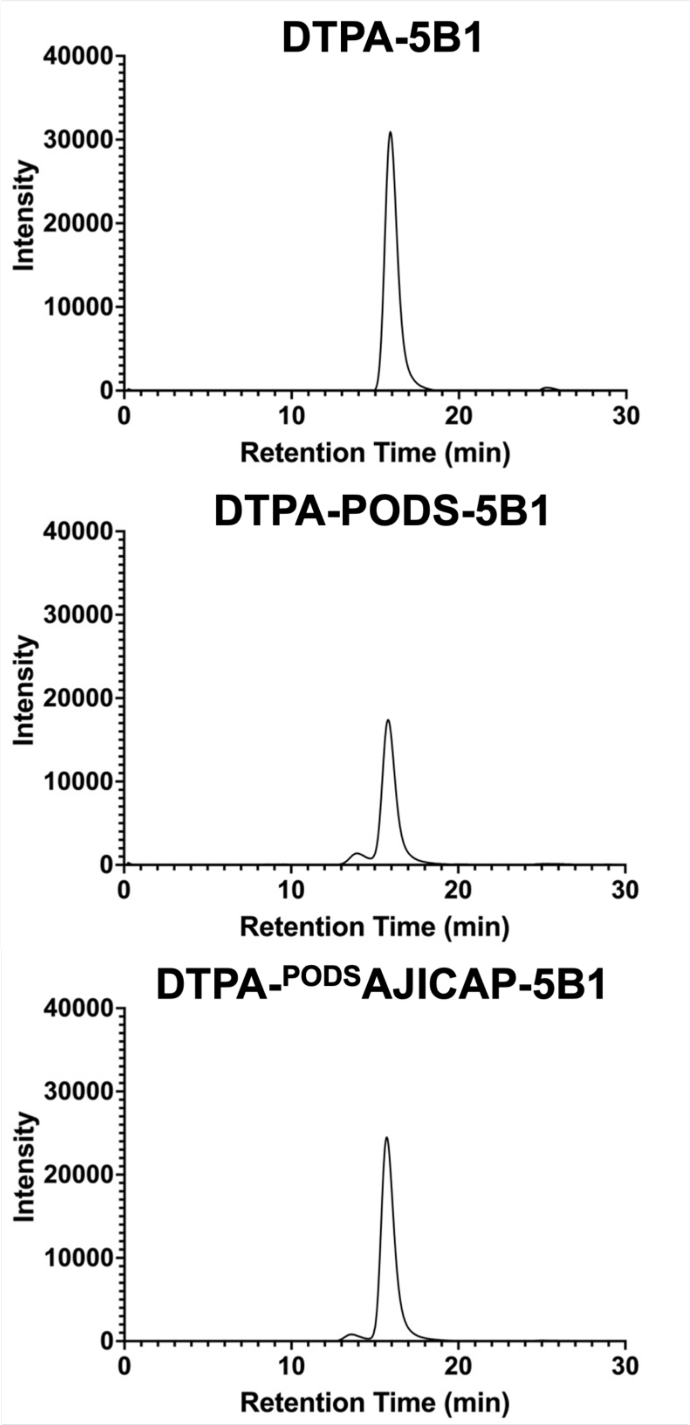

Figure S2. Size exclusion HPLC chromatograms for DTPA-5B1, DTPA-PODS-5B1, and DTPA-^{PODS}AJICAP-5B1.

Supplementary Tables

Sample	Average Mass (Da) n = 3	DOL (cargo/mAb)
5B1	148,476.7 ± 15.5	-
DTPA-5B1	150,526.8 ± 435.1	2.9 ± 0.6
DTPA-PODS-5B1	151,706.7 ± 130.5	2.8 ± 0.1
AJICAP-5B1	148,366.0 ± 50.5	-
DTPA-^{PODS}AJICAP-5B1	150,368.7 ± 63.1	1.8 ± 0.1

Table 1. MALDI-ToF mass spectrometry data used to determine the degree of labeling (DOL) of DTPA-PODS-5B1 and DTPA-^{PODS}AJICAP-5B1.

Radioimmunoconjugate	Specific Activity (mCi/mg)	Radiochemical yield (%)	Radiochemical Purity (%)
[^{177}Lu]Lu-DTPA-5B1	5	95.0	99.2
[^{177}Lu]Lu-DTPA-PODS-5B1	5	96.8	99.3
[^{177}Lu]Lu-DTPA-^{PODS}AJICAP-5B1	5	96.2	99.3

Table 2. Radiolabeling data — including specific activity, radiochemical yield, and radiochemical purity — for [^{177}Lu]Lu-DTPA-5B1, [^{177}Lu]Lu-DTPA-PODS-5B1, and [^{177}Lu]Lu-DTPA-^{PODS}AJICAP-5B1.

	48 h					
	[^{177}Lu]Lu-DTPA-5B1		[^{177}Lu]Lu-DTPA-PODS-5B1		[^{177}Lu]Lu-DTPA-^{PODS}AJICAP-5B1	
**Organ**	**%ID/g**	**Tumor:organ ratio**	**%ID/g**	**Tumor:organ ratio**	**%ID/g**	**Tumor:organ ratio**
**Blood**	3.1 ± 2.1	9.2 ± 7.8	11.8 ± 7.8	2.8 ± 2.2	7.1 ± 3.3	2.5 ± 2.3
**Tumor**	28.2 ± 14.6	1.0 ± 0.0	32.5 ± 14.1	1.0 ± 0.0	17.9 ± 14.0	1.0 ± 0.0
**Heart**	1.0 ± 0.6	28.9 ± 23.5	2.7 ± 0.4	12.0 ± 5.5	2.2 ± 0.9	8.0 ± 7.0
**Lungs**	1.7 ± 0.7	16.2 ± 10.4	4.1 ± 1.8	8.0 ± 5.0	3.6 ± 2.8	5.0 ± 5.4
**Liver**	12.8 ± 7.4	2.2 ± 1.7	4.3 ± 1.2	7.6 ± 3.9	3.3 ± 1.0	5.4 ± 4.5
**Spleen**	9.1 ± 6.5	3.1 ± 2.7	3.0 ± 1.3	10.7 ± 6.4	3.1 ± 1.2	5.8 ± 5.1
**Pancreas**	0.4 ± 0.3	68.2 ± 63.4	0.8 ± 0.3	39.6 ± 22.7	0.6 ± 0.3	28.9 ± 25.6
**Stomach**	0.7 ± 0.5	41.3 ± 36.9	0.8 ± 0.2	40.6 ± 21.6	0.6 ± 0.2	31.4 ± 26.2
**S. Intestine**	0.4 ± 0.2	67.8 ± 45.9	0.9 ± 0.4	34.9 ± 20.8	0.8 ± 0.1	23.6 ± 19.0
**L. Intestine**	0.4 ± 0.1	76.1 ± 40.6	0.6 ± 0.2	54.2 ± 28.8	0.5 ± 0.1	35.1 ± 28.4
**Kidneys**	2.0 ± 0.5	14.4 ± 8.2	3.4 ± 1.1	9.5 ± 5.1	2.9 ± 0.9	6.2 ± 5.3
**Ovaries/Uterus**	0.8 ± 0.5	37.7 ± 31.1	2.9 ± 2.8	11.4 ± 12.1	1.4 ± 0.4	12.6 ± 10.6
**Muscle**	0.3 ± 0.2	94.8 ± 82.4	0.7 ± 0.4	50.0 ± 40.4	0.5 ± 0.2	35.1 ± 31.6
**Bone**	1.0 ± 0.3	28.3 ± 16.5	5.7 ± 1.1	5.7 ± 2.7	1.6 ± 0.4	11.3 ± 9.2
**Skin**	2.9 ± 1.6	9.8 ± 7.5	4.7 ± 1.9	6.9 ± 4.1	3.7 ± 1.0	4.8 ± 4.0
**Tail**	0.9 ± 0.3	32.3 ± 20.5	1.7 ± 0.2	19.5 ± 8.8	1.2 ± 0.2	14.6 ± 11.5

	96 h					
	[^{177}Lu]Lu-DTPA-5B1		[^{177}Lu]Lu-DTPA-PODS-5B1		[^{177}Lu]Lu-DTPA-^{PODS}AJICAP-5B1	
**Organ**	**%ID/g**	**Tumor:organ ratio**	**%ID/g**	**Tumor:organ ratio**	**%ID/g**	**Tumor:organ ratio**
**Blood**	2.4 ± 3.1	8.2 ± 13.3	6.1 ± 2.3	6.4 ± 3.9	5.7 ± 0.4	5.7 ± 3.2
**Tumor**	19.3 ± 18.5	1.0 ± 0.0	39.4 ± 18.7	1.0 ± 0.0	32.1 ± 17.8	1.0 ± 0.0
**Heart**	0.8 ± 0.9	24.7 ± 36.1	2.0 ± 0.8	20.0 ± 12.6	1.7 ± 0.1	18.5 ± 10.2
**Lungs**	1.7 ± 2.5	11.2 ± 19.5	2.4 ± 1.0	16.2 ± 10.1	4.4 ± 0.8	7.4 ± 4.3
**Liver**	8.1 ± 2.8	2.4 ± 2.4	3.7 ± 2.2	10.8 ± 8.3	3.8 ± 0.6	8.4 ± 4.8
**Spleen**	5.4 ± 3.6	3.6 ± 4.2	3.1 ± 1.4	12.7 ± 8.3	3.9 ± 0.3	8.3 ± 4.6
**Pancreas**	0.4 ± 0.3	48.2 ± 61.4	0.9 ± 0.5	44.3 ± 33.1	0.6 ± 0.3	58.4 ± 45.0
**Stomach**	0.5 ± 0.3	35.7 ± 38.7	0.4 ± 0.7	91.6 ± 146.8	0.5 ± 0.1	61.8 ± 37.8
**S. Intestine**	0.5 ± 0.3	35.7 ± 39.5	0.6 ± 0.3	63.6 ± 39.4	0.5 ± 0.1	60.6 ± 37.0
**L. Intestine**	0.4 ± 0.2	49.5 ± 54.2	0.4 ± 0.2	89.6 ± 64.1	0.4 ± 0.1	89.3 ± 52.0
**Kidneys**	1.2 ± 1.1	15.7 ± 20.2	4.8 ± 2.0	8.2 ± 5.2	1.8 ± 0.8	17.5 ± 12.4
**Ovaries/Uterus**	0.9 ± 0.8	22.4 ± 29.3	1.1 ± 0.3	36.5 ± 20.5	1.6 ± 1.4	20.5 ± 21.1
**Muscle**	0.5 ± 0.6	41.9 ± 65.2	0.4 ± 0.5	112.6 ± 154.6	0.4 ± 0.2	89.3 ± 64.9
**Bone**	0.8 ± 0.3	24.1 ± 25.0	3.5 ± 1.6	11.4 ± 7.6	1.2 ± 0.4	27.9 ± 18.3
**Skin**	4.6 ± 5.5	4.2 ± 6.5	2.1 ± 1.2	18.8 ± 13.9	3.4 ± 0.9	9.5 ± 5.8
**Tail**	0.9 ± 0.6	21.0 ± 24.6	1.2 ± 0.3	32.8 ± 17.6	0.9 ± 0.1	35.3 ± 19.7

	144 h					
	[^{177}Lu]Lu-DTPA-5B1		[^{177}Lu]Lu-DTPA-PODS-5B1		[^{177}Lu]Lu-DTPA-^{PODS}AJICAP-5B1	
**Organ**	**%ID/g**	**Tumor:organ ratio**	**%ID/g**	**Tumor:organ ratio**	**%ID/g**	**Tumor:organ ratio**
**Blood**	1.8 ± 2.7	10.6 ± 16.3	3.3 ± 42.9	13.0 ± 6.2	2.1 ± 0.9	24.5 ± 15.6
**Tumor**	18.8 ± 5.0	1.0 ± 0.0	42.9 ± 13.7	1.0 ± 0.0	52.0 ± 24.5	1.0 ± 0.0
**Heart**	0.7 ± 0.8	27.7 ± 32.3	1.1 ± 0.3	40.1 ± 18.0	0.8 ± 0.4	61.9 ± 39.5
**Lungs**	3.0 ± 4.9	6.2 ± 10.2	1.8 ± 1.2	24.1 ± 17.9	2.5 ± 1.8	21.0 ± 17.9
**Liver**	7.2 ± 0.8	2.6 ± 0.7	2.5 ± 0.9	17.5 ± 8.7	4.3 ± 2.0	12.0 ± 8.0
**Spleen**	3.5 ± 0.9	5.3 ± 2.0	2.4 ± 0.8	17.7 ± 7.9	6.6 ± 4.3	7.8 ± 6.2
**Pancreas**	0.2 ± 0.2	81.9 ± 89.2	0.4 ± 0.1	113.0 ± 41.9	0.4 ± 0.1	140.4 ± 77.2
**Stomach**	0.4 ± 0.5	49.6 ± 64.7	0.4 ± 0.1	110.1 ± 38.1	0.3 ± 0.1	173.2 ± 98.8
**S. Intestine**	0.3 ± 0.2	69.7 ± 63.2	0.5 ± 0.1	93.4 ± 40.5	0.4 ± 0.2	120.8 ± 73.7
**L. Intestine**	0.3 ± 0.2	62.8 ± 41.0	0.5 ± 0.2	87.6 ± 51.4	0.3 ± 0.1	185.5 ± 103.5
**Kidneys**	1.1 ± 0.7	16.8 ± 11.0	2.2 ± 0.2	19.8 ± 6.7	1.7 ± 0.4	30.4 ± 16.3
**Ovaries/Uterus**	1.2 ± 1.0	16.4 ± 14.9	0.8 ± 0.8	51.7 ± 52.5	1.2 ± 0.5	41.9 ± 26.1
**Muscle**	0.4 ± 0.5	49.6 ± 61.4	0.3 ± 0.2	148.1 ± 88.3	0.3 ± 0.1	167.6 ± 93.0
**Bone**	0.6 ± 0.1	31.9 ± 9.9	5.3 ± 1.2	8.1 ± 3.2	1.3 ± 0.4	41.2 ± 22.7
**Skin**	2.6 ± 0.9	7.2 ± 3.2	1.5 ± 0.9	29.0 ± 19.2	1.6 ± 0.6	32.9 ± 20.4
**Tail**	0.9 ± 0.3	22.2 ± 10.5	0.9 ± 0.3	46.7 ± 19.9	0.7 ± 0.1	77.5 ± 39.5

Table 3. Biodistribution data (%ID/g values) and tumor-to-healthy organ activity concentration ratios at 48, 96, and 144 h after the administration of [^{177}Lu]Lu-DTPA-5B1, [^{177}Lu]Lu-DTPA-PODS-5B1, and [^{177}Lu]Lu-DTPA-^{PODS}AJICAP-5B1 to athymic nude mice bearing subcutaneous BxPC-3 xenografts.
